# A single exposure to brivaracetam or perampanel does not cause cell death in neonatal rats

**DOI:** 10.3389/fped.2024.1441891

**Published:** 2024-09-09

**Authors:** Eric Witherspoon, Nicholas Zuczek, Gabrielle Williams, Briana Bernstein, Anjik Ghosh, Marko Culjat, Suhasini Kaushal, Patrick A. Forcelli

**Affiliations:** ^1^ Department of Pharmacology & Physiology, Georgetown University, Washington, DC, United States; ^2^ Interdisciplinary Program in Neuroscience, Georgetown University, Washington, DC, United States; ^3^ Department of Neuroscience, Georgetown University, Washington, DC, United States

**Keywords:** developmental neurotoxicity (DNT), neonatal seizures, pregnancy, anti-seizure medication, apoptosis, programmed cell death

## Abstract

**Introduction:**

Exposure to a range of anti-seizure medications (ASMs) during early brain development adversely impacts neurodevelopmental outcomes in both animal models and in clinical studies. Many ASMs, including phenobarbital, phenytoin, valproate (VPA), and benzodiazepines, are associated with acute neurotoxicity (cell death), impaired synaptic development, and long-term behavioral changes following gestational or neonatal exposure in animals. This is mirrored in clinical studies which show lasting neurodevelopmental deficits following early-life or gestational exposure to these drugs. Brivaracetam (BRV) and perampanel (PER) are two newer generation anti-seizure medications and are of interest based on their mechanisms of action (SV2A modulator, AMPA antagonist, respectively), as other drugs with these mechanisms of action do not trigger acute neurotoxicity. Both BRV and PER show anti-seizure efficacy in developing animals, but potential neurotoxicity of these drugs is unexplored.

**Methods:**

To address this gap, we treated postnatal day (P)7 Sprague-Dawley rats with BRV (20, 40, 80 mg/kg) and PER (0.1, 0.9, 2.7 mg/kg), and assessed the induction of cell death across a range of vulnerable brain regions 24 h after exposure. Cell death was assessed using pathogreen staining.

**Results:**

In each of the regions examined (dorsal striatum, nucleus accumbens, motor cortex, cingulate cortex, lateral thalamus, septum, hippocampus), VPA, which served as a positive control, significantly increased cell death as measured by the numer of pathogreen positive cells. By contrast, neither BRV nor PER increased the number of pathogreen positive cells in any region examined.

**Discussion:**

Our results suggest that BRV and PER may have a positive safety profile–at least with respect to acute induction of cell death - and therefore may offer a safer option for the treatment of early life seizures.

## Introduction

The treatment of seizures during critical periods of brain development poses a unique set of challenges—both the treatment of neonatal seizures, and the treatment of pregnant women with epilepsy requires balancing *seizure control* with the potential for adverse effects of pharmacotherapy on the developing brain.

Many classic and first-line anti-seizure medications (ASMs) are associated with both acute and long-term toxicities (1, 2). One notably acute toxicity shared by many ASMs is the acute induction of cell death in postnatal day (P)7 rats. Valproate, phenobarbital, phenytoin, diazepam, clonazepam, lamotrigine, vigabatrin—as well as ethanol and anesthetic agents, all induce acute neurotoxicity after a single exposure at this age. The induction of neuronal apoptosis by these drugs occurs during a narrow postnatal window of brain development in rodents, with a peak around P7 (3–5). This timepoint, which corresponds to the peak of the brain growth spurt (6), models a period spanning from the third trimester through early infancy in humans.

Many of these drugs are also associated with impaired synaptic development, and long-term behavioral disruption (7–10). These findings have been mirrored in clinical studies, which show long-term alterations in brain volume (11), and neurodevelopmental delays following *in utero* or post-natal treatment (12, 13).

Fortunately, therapeutic agents like levetiracetam (LEV) have grown in use due to its favorable clinical safety and efficacy profile. Moreover, LEV is one of only a few ASMs that does not trigger cell death in the developing brain, suggesting that it may have a more benign safety profile (14). While many ASMs have been examined in preclinical assays for developmental neurotoxicity following acute drug exposure, many remain unexplored. Two drugs of considerable interest are brivaracetam and perampanel.

Brivaracetam, like levetiracetam, is an antagonist at the synaptic-vesicle 2A (SV2A) site (15). Moreover, brivaracetam has shown preclinical and clinical efficacy against neonatal seizures (16–18) making it a promising drug candidate. Perampanel is likewise of interest; it is a selective antagonist of α-amino−3-hydroxy-5-methyl-4-isoxazolepropionic acid (AMPA) receptors, and displays efficacy over a range of postnatal ages in rodents (16). It is currently approved for pediatric patients with focal onset seizures older than 4 years of age (19). While N-methyl-D-aspartate (NMDA) receptor antagonists are potent inducers of apoptosis in neonatal animals, other AMPA receptor antagonists *do not* induce cell death (4).

In the present study we sought to determine if *acute* exposure to brivaracetam or perampanel would induce cell death in the P7 rat brain. Whether perampanel and brivaracetam would share the neurotoxic profile described for many other ASMs (see above), or rather would match the safer profile seen for other drugs in their respective classes remains unknown. To address this gap, we treated P7 rat pups with brivaracetam or perampanel, and measured induction of cell death 24 h after treatment.

## Materials & methods

### Animals

Timed-pregnant Sprague-Dawley rats were acquired from Harlan/Envigo (Frederick, MD) on gestational day 15 and housed in the Department of Comparative Medicine (DCM) at Georgetown University. Animals were kept in a temperature-controlled environment at 21°C under a 12-hour light cycle (Lights on: 6:00–18:00 h). All procedures were performed in accordance with protocol (#2016-1306), which was approved by the Georgetown University Animal Care and Use Committee. The day of birth was defined as postnatal day (P) 0. On postnatal day (P) 7, pups were treated with drugs and euthanized 24 h later at postnatal day (P) 8. P7 rats were chosen as this age and model corresponds to a period spanning the late third trimester through early infancy in humans (6). Moreover, P7 also represents the developmental age where there is maximum proapoptotic effects induced by ASMs, anesthetics, and ethanol (3, 4, 20–22), and is the time that has been examined for essentially all of the ASMs that have been evaluated for pro-apoptotic effects. This allows for a direct comparison between our present findings and prior studies. All brain tissue was processed at the same time and treatments were counterbalanced across sex and within litters.

### Drugs

Vehicle solution was prepared using the following combination: methylparaben (0.2 mg/ml), citric acid (0.18 mg/ml), sodium citrate dihydrate (0.58 mg/ml), sodium carboxymethylcellulose (1 mg/ml), sucralose (8 mg/ml), sorbitol (48 mg/ml), and glycerin (30.4 mg/ml). Vehicle stock solution was then diluted with deionized water in a 1:4 ratio to maintain the osmolarity at <1,000 mOsm/dl.

Valproate was used as a positive control for the cell death assay (see below) as it is a well-established neurotoxic agent in the developing brain (3, 23). Valproate was diluted in vehicle solution at a concentration of 40 mg/ml to deliver a dose of 400 mg/kg intraperitoneally (ip), which is consistent with prior reports (3, 23, 24).

Brivaracetam was obtained as a stock solution from Union Chimique Belge (*UCB*; Brussels, Belgium) and diluted in vehicle to an initial concentration of 8 mg/ml. 4 and 2 mg/ml solutions of brivaracetam were then prepared using a 1:2 serial dilution to deliver final doses of 80, 40, and 20 mg/kg (ip) of drug.

Perampanel was obtain from Eisai (Woodcliff Lake, New Jersey) and diluted from 0.5 mg/mL stock solution into vehicle to make an initial concentration of .27 mg/ml drug. A 1:9 and 1:3 serial dilution was then prepared to make a final concentration of 0.09 and 0.01 mg/ml of perampanel, respectively. Final doses of perampanel were delivered at of 2.7, 0.9, and 0.1 mg/kg (ip) bodyweight. All drugs were administered intraperitoneally (ip) at a volume of 0.01 ml/g bodyweight and fell within the anticonvulsant dose range in neonatal rodents that we have previously reported (16).

The dose range we used for both brivaracetam and perampanel included a dose that was higher than the therapeutically relevant range by allometric scaling. We used the allometric scaling approach described in (25), which converts a rat dose (in mg/kg) to a human dose (in mg/kg) by dividing the animal dose by 6.2. For brivaracetam, our highest dose was 80 mg/kg. The human equivalent dose (HED) for this treatment was 12.9 mg/kg. Note that the label recommends 6 mg/kg/day in pediatric patients weighing less than 11 kg (26). Thus, our highest dose was twice the recommended maximum dose. For perampanel, our highest dose was 2.7 mg/kg. The HED for this treatment was 0.435 mg/kg. The label recommends a starting dose of 2 mg/day with a maximum of 12 mg/day (27). For an adult, the scaled dose would result in a dose of 26 mg/day. There are no established dosing guidelines for patients under the age of 12.

Thus, the high end of the dose range we selected for each drug represents a extreme dose and was chosen as the most robust test of induction of toxicity.

### Cell death

Twenty-four hours (24 h) following drug treatment, pups were anesthetized with an overdose of euthasol (40 mg/kg) and perfused with phosphate buffered saline followed by 4% paraformaldehyde. Brains were extracted and fixed in paraformaldehyde for 24 h before being transferred to a 30% sucrose solution for three days. The 24 h survival time is consistent with prior studies using late-stage markers of cell death such as fluorojade, TUNEL, and silver staining to detect drug-induced cell death in neonatal rats (3, 23, 28–30).

Following cryoprotection in sucrose, brains were sliced coronally at 35 microns thick sections using a Leica CM1800 cryostat.

Pathogreen histofluorescent dye (Biotin, catalog #: 80027) was applied to detect degenerating neuronal cell bodies. The procedure consisted of several steps involving submerging slides in decreasing concentrations of ethanol (80% and 70%) followed by submersion of slides in diH_2_0 for 2 min. Slides were then placed in 0.06%KmO_4_ for roughly 10mins followed by an additional submersion in diH_2_0 for 2 min, where the diH_2_0 step was done twice. Pathogreen was made according to the manufacturer's protocol (PI-80027) at a 1:1,000 mix and applied to slides containing tissue samples for roughly 10mins. Slides were again washed with diH_2_0 and air-dried prior to imaging.

Photomicrographs were taken at 10× magnification from 3 consecutive brain regions using a Nikon 80i light microscope. The neonatal rat brain atlas (31) was used as a guide to define the following brain regions of interest (ROI): striatum, septum, cingulate cortex, nucleus accumbens, motor cortex, lateral thalamus, and hippocampus. These regions were selected based on prior studies from our group and others (3, 32) demonstrating vulnerability to ASM induced cell death.

QuPath v0.3.2 software was used to automate counting of pathogreen-positive cells and the total number of positive cells was averaged across the three consecutive brain regions imaged. Analysis was also performed using 2 observers who were blind to treatment groups.

### Statistics

GraphPad Prism 9 (GraphPad Software; La Jolla, Ca) was used for statistical analysis of histology data. To assess if the data were normally distributed across groups, we used the Shapiro Wilk normality test. The number of pathogreen positive cells were analyzed using the Analysis of Variance (ANOVA) followed by Holm-Sidak multiple comparisons test. *P*-values <0.05 were considered statistically significant.

## Results

To evaluate the safety profile of brivaracetam and perampanel with respect to induction of cell death in the neonatal rodent brain, we examined cortical, subcortical, and limbic regions.

**Figure 1:**
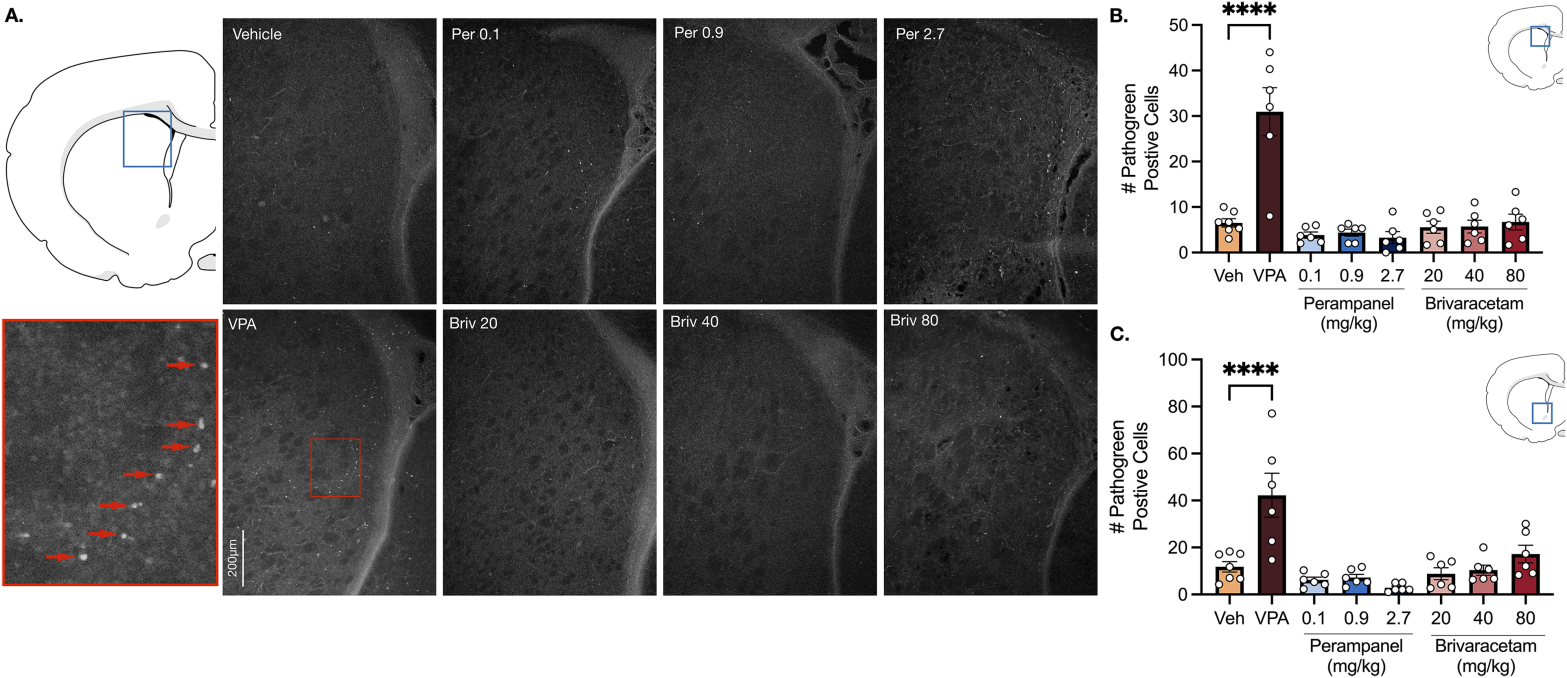
Exposure to valproate (VPA) but not brivaracetam (BRIV) or perampanel (PER) induces cell death in basal ganglia regions. **(A)** Photomicrographs displaying pathogreen-positive cell bodies in the striatum of postnatal day 8 rats. Blue box = representative region of interest of captured photomicrograph; red box = magnified striatal photomicrograph with positive-pathogreen cell bodies indicated by red arrows **(B)** mean (± SEM) number of pathogreen-positive cells. Valproate-treated rodents displayed higher levels of neurodegeneration compared to vehicle-treated groups (*p* < 0.0001, Holm-Sidak). Scale bar = 200 μm. (Veh *n* = 7; VPA *n* = 6; PER 0.1 mg/kg *n* = 6; PER 0.9 mg/kg *n* = 6; PER 2.7 mg/kg *n* = 6; BRIV 20 mg/kg *n* = 6; BRIV 40 mg/kg *n* = 6; BRIV 80 mg/kg *n* = 6) **(C)** Mean (± SEM) number of pathogreen-positive cells in nucleus accumbens. VPA-treated animals had significantly higher levels of degenerating neurons compared to vehicle-treated groups (*p* < 0.0001, Holm-Sidak). Scale bar = 200 μm. (Veh *n* = 7; VPA *n* = 6; PER 0.1 mg/kg *n* = 6; PER 0.9 mg/kg *n* = 6; PER 2.7 mg/kg *n* = 6; BRIV 20 mg/kg *n* = 6; BRIV 40 mg/kg *n* = 6; BRIV 80 mg/kg *n* = 6).

Vehicle treated rats displayed a low baseline level of pathogreen-positive cells in basal ganglia sub-regions (i.e., striatum, nucleus accumbens) 24 h after pretreatment (Figure 1). As expected, when pups were treated with valproate (400 mg/kg; positive control), the number of pathogreen positive cells within the striatum was significantly elevated (Figure 1B, *P* = 0.0001, Holm-Sidak; 4.7-fold increase).

Analysis of variance (ANOVA) revealed a main of effect of drug (F_7,41_ = 18.05, *P* = 0.0001), which was driven by the valproate condition. No significant difference was observed after exposure to perampanel (0.1, 0.9, 2.7 mg/kg) or brivaracetam (20, 40, 80 mg/kg) compared to vehicle treated animals (Figure 1B, Ps > 0.86, Holm-Sidak).

Similar to the striatum, we found low levels of pathogreen-positive cells in the nucleus accumbens after vehicle treatment (Figure 1C). When postnatal-day (P) 7 pups were treated with valproate (400 mg/kg), we observed a significant (3.6-fold) increase compared to control groups (Figure 1C, *P* = 0.0001, Holm-Sidak). While analysis of variance (ANOVA) revealed a main effect of drug (F_7,41_ = 10.23, *P* = 0.0001), again driven by the valproate condition, neither perampanel nor brivaracetam increased the number of pathogen positive cells relative to vehicle controls, at any dose tested (Figure 1C, Ps > 0.47, Holm-Sidak).

**Figure 2:**
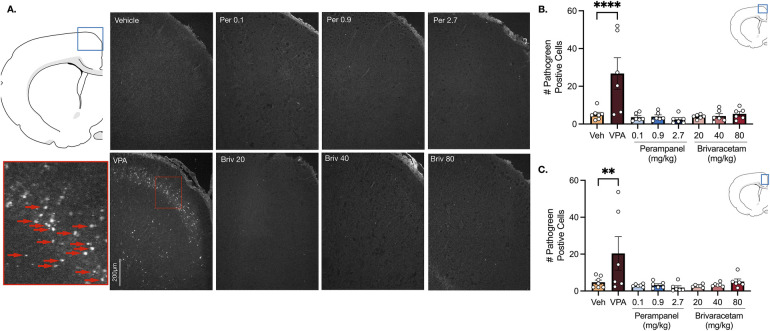
Exposure to valproate (VPA) but not brivaracetam (BRIV) or perampanel (PER) induces cell death in cortical regions. **(A)** Photomicrographs displaying pathogreen-positive cell bodies in the motor cortex of postnatal day 8 rats. Blue box = representative region of interest of captured photomicrograph; red box = magnified photomicrograph with positive-pathogreen cell bodies indicated by red arrows **(B)** mean (± SEM) number of pathogreen-positive cells in motor cortex. VPA-treated animals displayed significantly higher levels of pathogreen positive compared to vehicle-treated groups (*p* < 0.0001, Holm-Sidak). Scale bar = 200 μm. (Veh *n* = 7; VPA *n* = 6; PER 0.1 mg/kg *n* = 6; PER 0.9 mg/kg *n* = 6; PER 2.7 mg/kg *n* = 6; BRIV 20 mg/kg *n* = 6; BRIV 40 mg/kg *n* = 6; BRIV 80 mg/kg *n* = 6). **(C)** Mean (± SEM) number of pathogreen-positive cells in the cingulate cortex. VPA-treated animals had significantly higher levels of pathogreen-positive compared to vehicle-treated groups (*p* = 0.0081, Holm-Sidak). Scale bar = 200 μm. (Veh *n* = 7; VPA *n* = 6; PER 0.1 mg/kg *n* = 6; PER 0.9 mg/kg *n* = 6; PER 2.7 mg/kg *n* = 6; BRIV 20 mg/kg *n* = 6; BRIV 40 mg/kg *n* = 6; BRIV 80 mg/kg *n* = 6).

In the motor cortex, vehicle treated animals displayed low levels of neuronal cell death (Figure 2B). Valproate induced a 5.2-fold increase in pathogreen positive cells (Figure 2B, F_7,41_ = 6.94, *P* = 0.0001; *P* = 0.0001, Holm-Sidak), an effect that was not observed after treatment with either perampanel or brivaracetam (Figure 2B, Ps > 0.99; Holm-Sidak corrected). We found a similar pattern in the cingulate cortex (Figure 2C). Exposure following valproate pretreatment revealed a 4.3-fold increase in cell death (*P* = 0.0081, Holm-Sidak), while no significant difference was observed after exposure to perampanel or brivaracetam (Figure 2C, Ps > 0.98, F_7,41_ = 4.56, Holm-Sidak).

**Figure 3:**
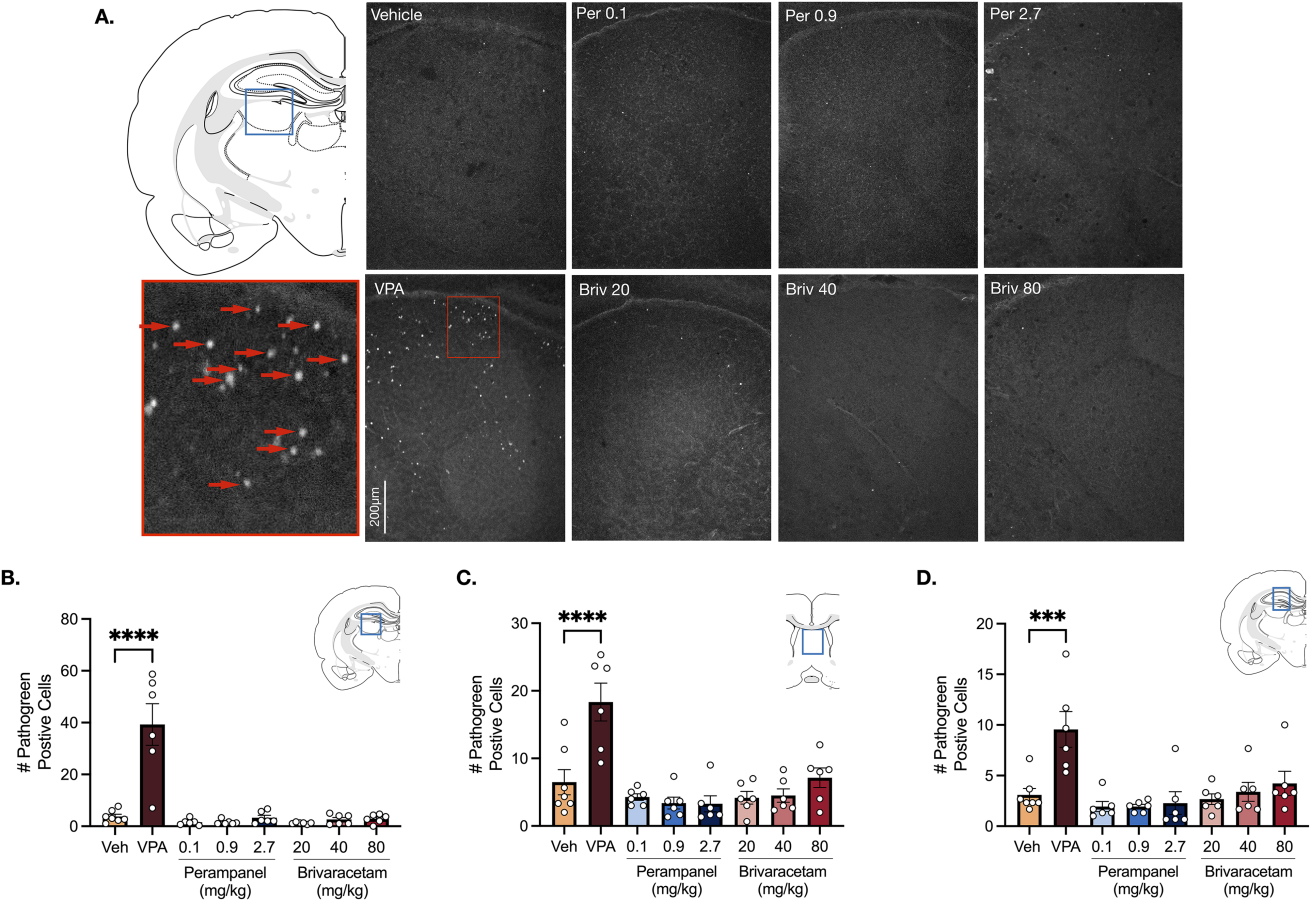
Exposure to valproate (VPA) but not brivaracetam (BRIV) or perampanel (PER) induces cell death in limbic and subcortical regions. **(A)** Photomicrographs displaying pathogreen-positive cell bodies in the thalamus of postnatal day 8 rats. Blue box = representative region of interest of captured photomicrograph; red box = magnified photomicrograph with positive-pathogreen cell bodies indicated by red arrows. **(B)** Mean (± SEM) number of pathogreen-positive cells in the lateral thalamus. VPA-treated animals had significantly higher levels of pathogreen-positive cells compared to vehicle-treated groups (*p* < 0.0001, Holm-Sidak). Scale bar = 200 μm. (Veh *n* = 7; VPA *n* = 6; PER 0.1 mg/kg *n* = 6; PER 0.9 mg/kg *n* = 6; PER 2.7 mg/kg *n* = 6; BRIV 20 mg/kg *n* = 6; BRIV 40 mg/kg *n* = 6; BRIV 80 mg/kg *n* = 6). **(C)** Mean (± SEM) number of pathogreen-positive cells in the septum. VPA-treated pups had significantly higher levels of pathogreen positive cells compared to vehicle-treated groups (*p* < 0.0001, Holm-Sidak). Scale bar = 200 μm. (Veh *n* = 7; VPA *n* = 6; PER 0.1 mg/kg *n* = 6; PER 0.9 mg/kg *n* = 6; PER 2.7 mg/kg *n* = 6; BRIV 20 mg/kg *n* = 6; BRIV 40 mg/kg *n* = 6; BRIV 80 mg/kg *n* = 6). **(D)** Mean (± SEM) number of pathogreen-positive cells in hippocampus. VPA-treated pups had significantly higher levels of pathogreen positive cells compared to vehicle-treated groups (*p* = 0.0001, Holm-Sidak). Scale bar = 200 μm. (Veh *n* = 7; VPA *n* = 6; PER 0.1 mg/kg *n* = 6; PER 0.9 mg/kg *n* = 6; PER 2.7 mg/kg *n* = 6; BRIV 20 mg/kg *n* = 6; BRIV 40 mg/kg *n* = 6; BRIV 80 mg/kg *n* = 6).

Analysis of variance (ANOVA) revealed a main effect of drug treatment in the lateral thalamus (Figure 3B, F_7,41_ = 20.69, *P* < 0.0001), septum (Figure 3C, F_7,41_ = 10.70 *P* < 0.0001) and hippocampus (Figure 3D, F_7,41_ = 6.12, *P* < 0.0001). Holm-Sidak multiple comparisons test confirmed that valproate (400 mg/kg) significantly increased cell death in all limbic (i.e., hippocampus, septum) and subcortical (i.e., lateral thalamus) regions compared to vehicle-treated controls (Figure 3, Ps for the Holm Sidak here). Neither perampanel (0.1, 0.9, 2.7 mg/kg) or brivaracetam (20, 40, 80 mg/kg) significantly increased cell death in the lateral thalamus (Figure 3B, Ps > 0.99, Holm-Sidak), septum (Figure 3C, Ps > 0.57, Holm-Sidak) or hippocampus (Figure 3D, Ps > 0.95, Holm-Sidak) compared to vehicle treated controls.

## Discussion

Here, we report a lack of significant cell death induced by brivaracetam and perampanel in neonatal (P7) rodents. Consistent with prior reports, we found that valproate, a prototypical antiseizure drug, induced robust cell death in limbic, basal ganglia, cortical, and subcortical regions. By contrast, brivaracetam and perampanel did not elicit the same effect at doses that are effective at suppressing seizures in early stages of postnatal development (16).

Brivaracetam, like levetiracetam also selectively binds to synaptic vesicle 2A (SV2A) sites, resulting in a reduction in excitatory neurotransmitter release during enhanced neuronal excitability (33). Our group as well as others have extensively reported that levetiracetam does not trigger cell death in neonatal rodents (14, 23, 34).

Similarly, while it is well established that drugs that target glutamatergic neurotransmission (i.e., particularly NMDA receptors) display efficacy against neonatal seizures (35), the impact of NMDA receptor antagonist on neuronal apoptosis in neonatal rodents is unfavorable (30, 36). For example, MK801—a common NMDA antagonist—protects against kainic-acid induced seizures, but also induces significant apoptosis in cortical and subcortical regions in the immature brain (30, 37). By contrast, other AMPA receptor antagonists do not induce cell death in neonatal rodents (38, 39).

In some respects, our data while encouraging, are not surprising. While mechanism or drug-class specific safety/toxicity profiles would expedite neurotoxicity assessment, unfortunately, drug mechanism of action is not a perfect predictor of neurotoxic potential. For example, voltage gated sodium channel blockers display wide-ranging profiles with respect to cell death in neonatal animals. While phenytoin triggers cell death at therapeutic doses, lamotrigine and carbamazepine only triggers cell death at supratherapeutic doses, or, in the case of lamotrigine, when given as part of a polytherapeutic cocktail.

Brivaracetam has been only sparsely investigated in preclinical models of neonatal seizures. However, pharmacokinetic studies show that brivaracetam has a greater lipophilicity and is roughly 20 times more potent than its analog levetiracetam (15, 40). In the clinical setting, brivaracetam displays efficacy coupled with a favorable side effect profile at nearly 10 times lower than the concentration of levetiracetam (41, 42).

In the present study, we examined only one marker of cell death, pathogreen histoflourescence. This approach, which is similar to fluorojade staining (43) marks degenerating neurons. We and others have previously used a wide range of methods for detecting cell death following neonatal anti-seizure medication exposure, including TUNEL staining, Amino Cupric Silver staining, morphology by electron microscopy, activated caspse-3 immunohistochemistry, and fluorojade staining. The profile across all of these approaches has been uniformly consistent, suggesting that these findings are not specific to a particular method of cell death detection.

One obvious limitation of the present study is that while there is a lack of acute cell death following brivaracetam and perampanel exposure, it has yet to be determined what impact chronic exposure to these ASMs has on neurotoxicity. Whether either BRIV or PER would display cell death with even higher doses, or as part of a polytherapy cocktail remains to be explored. Moreover, cell death likely only represents the “tip of the iceberg” in terms of developmental neurotoxicity. We have previously found that drug doses that are below the threshold for induction of cell death still can impair synaptic development (8, 36). Moreover, many ASMs produce long-term effects on behavior following early life exposure in rodent models (9, 36, 44). Whether or not brivaracetam or perampanel would show a safe profile with respect to synaptic or behavioral functions remains unknown. However, at least with respect to the induction of cell death, which is observed across a wide range of ASMs, brivaracetam and perampanel have a safer profile. These drugs merit further exploration for the treatment of neonatal seizures and seizures in pregnant women with epilepsy, including further evaluation of long-term outcomes following drug exposure.

## Conclusion

Here we find that perampanel and brivaracetam *do not* increase cell death in neonatal rats, even at doses that extend into the supratherapeutic range. This finding is strikingly different from many other ASMs, but mirrors the established safety profile of AMPA antagonists and SV2A modulators in the developing brain. These data suggest promise for these drugs for use during critical periods of brain development, and open the door to studies examining longer term-exposure as well as behavioral outcomes. If these drugs continue to display a favorable toxicity profile, clinical examination of them for use in neonates and pregnancy may be warranted.

## Data Availability

The raw data supporting the conclusions of this article will be made available by the authors, without undue reservation.
